# Revisiting the Role of Natural Killer Cells in Non-Alcoholic Fatty Liver Disease

**DOI:** 10.3389/fimmu.2021.640869

**Published:** 2021-02-18

**Authors:** María Luz Martínez-Chantar, Teresa C. Delgado, Naiara Beraza

**Affiliations:** ^1^Liver Disease Laboratory, Center for Cooperative Research in Biosciences (CIC bioGUNE), Basque Research and Technology Alliance, Derio, Spain; ^2^Centro de Investigación Biomédica en Red de Enfermedades Hepáticas y Digestivas (CIBERehd), Derio, Spain; ^3^Gut Microbes and Health Institute Strategic Programme, Food Innovation and Health Institute Strategic Programme, Quadram Institute Bioscience, Norwich Research Park, Norwich, United Kingdom

**Keywords:** NK cells, NAFLD, NASH, metabolism, obesity

## Abstract

Non-Alcoholic Fatty Liver Disease (NAFLD) is the most common form of chronic liver disease. The histological spectrum of NAFLD ranges from simple steatosis to chronic inflammation and liver fibrosis during Non-Alcoholic Steatohepatitis (NASH). The current view is that innate immune mechanisms represent a key element in supporting hepatic inflammation in NASH. Natural Killer (NK) cells are lymphoid cells and a component of the innate immune system known to be involved in NASH progression. Increasing evidence has shed light on the differential function of circulating and tissue-resident NK cells, as well as on the relevance of metabolism and the microenvironment in regulating their activity. Here, we revisit the complex role of NK cells as regulators of NASH progression as well as potential therapeutic approaches based on their modulation.

## Introduction

Non-Alcoholic Fatty Liver Disease (NAFLD) is the most common form of chronic liver disease in western countries and affects over 25% of the population globally (1). NAFLD pathophysiological effects extend beyond liver-related morbidity and mortality as NAFLD also increases the risk of T2DM, cardiovascular (CVD) and cardiac diseases, and chronic kidney disease (CKD) (1). NAFLD comprehends a group of conditions that share, as a common feature, the accumulation of fat in the liver as a result of non-alcoholic and non-viral causes. The histological spectrum of NAFLD ranges from simple steatosis, usually considered rather benign, to Non-Alcoholic Steatohepatitis (NASH), characterized by lobular inflammation; patients with NASH are more likely to progress to advanced fibrosis, cirrhosis (2) and eventually to hepatocellular carcinoma (HCC) (3).

## Inflammation as the Hallmark of the Progression From Non-Alcoholic Fatty Liver Disease (NAFLD) to Non-Alcoholic Steatohepatitis (NASH)

Chronic inflammation is a hallmark of NASH, characterized by profound dysregulation of the different innate and adaptive immune cell compartments as reviewed elsewhere (4, 5).

Briefly, NASH is characterized by robust recruitment of immune cells into the liver where they become activated (5). While a dysregulated immune response can further exacerbate liver disease [reviewed in (6)], the inflammatory response occurring early during liver injury may be important for healing and tissue repair (7, 8), adding complexity to the implication of immune cells in the pathogenesis of NAFLD/NASH. Single-cell-based transcriptomics studies showed that immune cellular heterogeneity and dysregulation are concomitant with the pathogenesis of NAFLD/NASH in patients (9) and in diet-induced obesity (DIO)-NASH mice (10, 11). The frequencies of different immune cell compartments are altered during NAFLD and the specific role of some of these immune cell populations in promoting NASH has been well-established. For example, KCs are enriched in NASH livers, where produce inflammatory cytokines and facilitate the development of fibrosis and HCC [reviewed in (12, 13)]. Likewise, neutrophils, are recruited into the liver during NASH in response to several chemokines contributing to the progression of NASH and pathogenesis of HCC (14). On the other hand, CD4+ T cells and regulatory T (Tregs) are decreased in experimental NASH, whereas IL17-producing T cells increased and CD8+ T cells were activated (15). In agreement with the different frequencies observed, T helper 22 (Th22) cells, a subset of CD4+ T cells, and regulatory T (Treg) cells appear to hamper NAFLD progression whereas Th17 and cytotoxic T (Tc) cells further promote liver injury and fibrosis progression [reviewed in (16)].

The importance of other populations of immune cells, such as DC, NKT cells, B cells, and NK cells in promoting NASH is less clear and is still under debate.

## Natural Killer Cells: a Heterogenous Multifunctional Population

Although adaptive immunity promotes liver inflammation in NAFLD, current views suggest that innate immune mechanisms are a key element supporting hepatic inflammation in NASH (17). NK cells, a component of the innate immune system, were first functionally identified in 1975 as a unique group of lymphocytes based on the presence of distinctive cytoplasmic granules (18–21). Functionally, NK cells kill their targets via secretion of lytic granules that contain pore-forming perforin and apoptosis-inducing granzymes (22, 23). In humans, there are two subsets of NK cells based on the relative expression of specific surface markers: the CD56^dim^CD16^+^ phenotype and the CD56^bright^CD16^dim−/+^ phenotype. CD56^dim^ NK cells are important for immediate cytotoxic killing of target cells, whereas CD56^bright^ NK cells require upregulation of the cytotoxic machinery before achieving cytotoxic potential (24). NK cells originate in the bone marrow and undergo a complex maturation process during which they acquire their effector functions; they then disperse and are present throughout lymphoid and non-lymphoid tissues. The lung has the highest frequency of NK cells amongst its lymphocytes, followed by the liver, peripheral blood, spleen, bone marrow, lymph nodes, and thymus.

Hepatic NK cells, originally called “Pit cells,” are situated inside the sinusoidal lumen, adhering to endothelial and KCs; morphologically they are defined as large granular lymphocytes (LGLs) and functionally as hepatic (liver-associated) NK cells (25). Human hepatic NK cells are a heterogeneous population, phenotypically and functionally, and include liver-resident NK cells (CCR5^+^CXC^R+^CD69^+^), memory-like NK cells (CXCR6^+^CD94/NKG2C^+^) and transient conventional NK cells mainly represented by recirculating NK cells (predominantly CD56^dim^CD16^+^ NK cells) in the liver blood system [reviewed in (26)]. While recirculating NK cells play a major role in host rejection of tumors and virus-infected cells (27); the liver-resident NK cells have an increased killing activity, express higher levels of cytotoxic mediators (e.g., IFNγ and TNF), and expression of CD69; an acute activation marker expressed transiently on recently activated lymphocytes. Memory-like NK cells, show unique adaptive traits and hapten-specificity, indicating that NKs can mediate long-lived, antigen-specific adaptive recall responses (28). However, the existence of a specific viral-antigen recognized by a given NK receptor expressed on human memory-like NK cells has not be yet demonstrated.

As key components of the liver innate immune system, hepatic NK cells can kill pathogens, tumor cells, stressed hepatocytes and HSCs, either directly or indirectly through the production of cytokines. NK cells can also target DCs, KCs, T cells, B cells, and endothelial cells through innate immunorecognition or by producing cytokines [e.g., IFN-gamma (IFNγ), tumor necrosis factor (TNF), interleukin-10 (IL-10)], chemokines and growth factors. The ability of hepatic NK cells to kill target cells is regulated by opposing signals from inhibitory and stimulatory receptors on their surfaces [reviewed in (29, 30)].

In the last years, NK cells have been described as important intermediates in several types of liver disease, including viral hepatitis, where they have a functional dichotomy featuring conserved or seldom enhanced cytolytic activity, together with dysfunctional cytokine production contributing to the persistence of the virus (31). NK cells can modulate drug-induced liver injury by direct interaction with hepatocytes resulting in cytotoxicity and IFNγ production (32). NKs are also relevant in liver cancer, where very recently the presence and dysfunction of liver-resident NK cells was reported and could therefore provide a new strategy for immune checkpoint-based targeting (33). Herein, we revisit the important role of NK cells in the pathogenesis and progression of NASH.

## Natural Killer Cells in Non-Alcoholic Fatty Liver Disease (NAFLD): Frequencies and Activation

To date, the presence, regulation and function of NK cells during NAFLD remains controversial (Table 1 for a summary of major findings to date). In recent years, several studies have found that the frequency of circulating NK cells in obese individuals is lower than in lean individuals, both for adults (36, 39) and children (38). Reduced frequency was associated with dysfunctional activity and characterized by lower levels of IFNγ (39) and reduced cytotoxic activity, with less granzyme B and perforin production and a reduced capacity to kill tumor cells (36, 38, 39). In line with this, circulating NK cells from obese patients appeared activated, with higher CD69^+^ expression but showed reduced cytokine production and cytotoxicity than cells from lean patients (35). Most recently, NAFLD patients were shown to have a lower frequency of CD56^dim^ NK cells with lower expression of the activating receptor NKG2D compared with healthy individuals (9). Comparable results were described in murine models of obesity where the percentage and total numbers of circulating and tissue-resident NKs were reduced in obese db/db mice deficient in the leptin receptor, compared with controls (45).

**Table 1:** NK cells characteristics and activity during human and experimental NAFLD/NASH.

**Human NAFLD/NASH**	**Site of detection**	**Changes in numbers/frequency**	**Activation/cytotoxicity**	**References**
NASH/NAFLD	Blood	Total numbers and frequencies not different.	Increased NKG2D in CD56bright/CD56dim	(34)
NASH obesity	Blood	Increase numbers rather than percentages.	Activated (high CD69, granzyme B) but less cytotoxic.	(35)
Obese	Blood	Significantly decreased NK cell levels.	NK function compromised	(36)
NAFLD/Fibrosis (Obese)	Blood	NK cell levels were significantly elevated in F4 subjects	Loss of tumor killing function.	(37)
NAFLD children (obese)	Blood	Decrease in NK cell frequency and absolute numbers	Increased activation with loss of function. Less cytotoxic (granzyme and perforin) but same IFNg	(38)
Obese/insulin resistant	Blood	Lower frequency of circulating NK cells (CD3–CD56+)	Lower tumor killing capacity. Less cytotoxic function (IFN-γ)	(39)
NAFLD	PBMC	Lower NK CD56dim with less NKG2D in PMBCs Liver; decreased NK56bright but increased CD56dim Fibrosis negatively correlated with liver total NKs	Less NKG2D	(9)
NAFLD	liver	Decreased CD56 bright but increased CD56dim	Less NKG2D Fibrosis negatively correlated with liver total NKs	(9)
Obese/insulin resistant	Blood	No differences in CD56 bright and dim numbers.	Increased IL6Ra expression. Increased myeloid differentiation and inflammasome related genes	(40)
NAFLD	Liver		Lower cytotoxic (decreased ability to degranulate)	(41)
NASH obese vs. healthy	Live	Lower numbers during NAFL Increased numbers in NASH Whereas NK cell numbers were NAFL patients revealed lower NKG2D mRNA transcription levels than patients with NASH	Lower NKG2D mRNA in NAFL Higher NKG2D and MICA/B in NASH.	(42)
**NAFLD/NASH Animal model**	**Site of detection**	**Changes in numbers/frequency**	**Activation/cytotoxicity**	**References**
NASH (with weight loss) MCD (8w)	Liver	Enrichment of NK via CXLC10	Increased activation (IFNg, TGFb IL10). Lower proliferation.	(43)
NAFLD (obese) High Fat/High Sugar diet (24 w)	Spleen/liver	No change in numbers	Less cytotoxic (less perforin in liver, not observed in spleen)	(41)
NASH (with weight loss) MCD (17 days)	Liver	No changes in frequency of DX5(+)NKp46(+) Low numbers of CD49a+NK1+ILC1	Increased activation (NKG2D). No changes in cytotoxicity. Polarizing Mϕ toward M1-like phenotypes via IFNg but not granzyme	(44)
Obese/insulin resistant HFD (16w)	Perigonadal adipose tissue	Increased NK cells numbers.	Increased IL6Ra expression. Increased myeloid differentiation/inflammation	(40)
Obese/insulin resistant HFD (16w)	blood	No difference in NK cells numbers.	Increased IL6Ra expression. Increased myeloid differentiation/inflammation	(40)
Obese/insulin resistant HFD (16w)	liver	No difference in NK cells numbers.	Increased IL6Ra expression. Increased myeloid differentiation/inflammation	(40)
Obese db/db mice (Leptin receptor KOs)	Blood, spleen, liver and lung	Decreased numbers and percentage	Less cytotoxic (leptin dependent).	(45)
NAFLD IL15 KO mice on HFD (16w)	Liver	Increased numbers on HFD but frequency comparable WT vs KO	IL15 KO have lower chemokine production.	(46)
Spontaneous NASH GNMT KO mice	Liver/Spleen	Decreased numbers	More cytotoxic, more activated with high TRAIL expression.	(47)

In apparent contradiction, other studies described no changes in either frequency or absolute numbers of circulating CD56^bright^ and CD56^dim^ NK cells in obese, NAFL, or NASH patients (34, 40), and similar cytokine production and cytotoxic activity (34). Interestingly, while the frequencies of CD56^bright^ and CD56^dim^ NK cells were maintained within the different treatment groups, differences in the phenotype/cytokine receptors were found. Thus, CD3^−^CD56^+^ NK cells from obese individuals (and in animal models of obesity and insulin resistance) had significantly higher expression levels of the interleukin-6 receptor (IL6R) (40). Likewise, despite similar frequencies, NK cells from NASH patients had higher levels of NKG2D than NK cells from healthy individuals, while no differences were observed in NAFLD patients (34). Of relevance, liver fibrosis is a key determinant for activation of circulating NK cells. Thus, CD56^dim^ NK cells were more cytotoxic in patients with low fibrosis scores while the tumor-cell-killing capacity of CD56^dim^ cells was significantly reduced in patients with high fibrosis scores (37). Differences in disease stage in the human cohorts studied (advanced NASH with fibrosis vs. NAFLD), could partly explain differential expression of NKG2D and circulating NK cell frequency.

NAFLD is a multisystemic disease and other organs besides the liver are involved in this pathology, e.g., the adipose tissue. Both CD56^bright^ and CD56^dim^ NK cells are present in adipose tissue (with CD56^bright^ being enriched) and are characterized by having distinct homing markers compared to blood NKs (e.g., CD25, CD69, and CD49a) during human and murine NASH (48, 49). The frequency and activity of NK cells in adipose tissue was comparable in healthy, NAFLD and NASH patients (with mild phenotypes; not including high BMI and severe cirrhosis) (34). However, studies in mice have shown increased accumulation of NK cells in perigonadal adipose tissue after feeding with a high-fat diet (HFD) that present a different transcriptional profiling, including increased IL6Ra and myeloid markers; Csf1 (40). The inhibition on these myeloid-related pathways significantly reduced obesity and insulin resistance, supporting that the tissue-specific characteristic of adipose NK cells contributes to the progression of NASH (40). The relevance of the NK cells-macrophages crosstalk was further supported by Wensveen and colleagues that showed increased expression of NK cell receptor NCR1 (NKp46)-activating ligands on adipocytes in mice after feeding with an HFD. This activated proliferation of NK cells and increased production of IFNγ, which further supported the infiltration and activation of macrophages in this tissue, key to maintain the systemic inflammatory status and insulin resistance characteristic of NASH (50). Likewise, the depletion of the transcription factor E4bp4 in NK cells led to the reduction of adipose tissue macrophages in obese mice, further underlining the relevance of the NK cells-macrophage interaction in contributing to obesity and insulin resistance (48).

**Figure 1:**
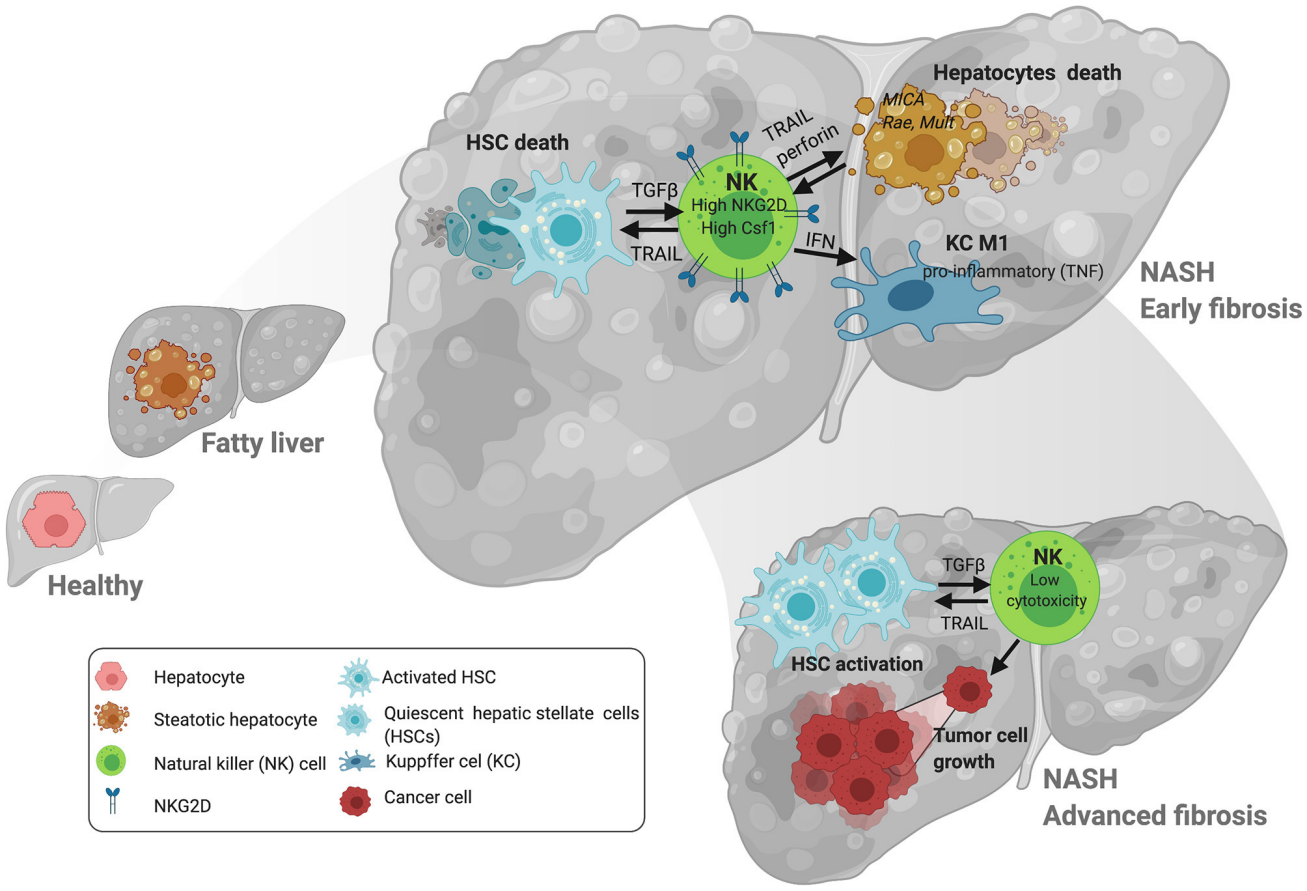
NK cell interactions with liver cells during NAFLD/NASH progression. At early stages of NAFLD, NK cells promote TRAIL-mediated death of hepatic stellate cells (HSC). NK cells promote the activation of other immune cells in the liver including macrophages via IFN-γ. Lipid accumulation in hepatocytes contributes to the expression of stress ligands that may promote the upregulation of the activating receptor NKG2D. This is relevant at later stages of NASH where increased activation of NKG2D by ligands including MICA (in patients) promote activation of NK cells. Loss of cytotoxic and tumoricidal activity in NK cells may contribute to the progression of hepatocellular carcinoma (HCC) at later stages of the disease. Figure created with BioRender.com.

Similarly to what was described in the adipose tissue, several studies found comparable NK frequencies in the livers of obese and lean patients (41) and in mice during experimental NASH, though liver NK cells exhibited increased cytokine (IFNγ) production (44) but reduced cytotoxic capacity, which was negatively correlated with disease severity (41) (Figure 1). In contrast, enrichment of NK cells in the liver was observed after consumption of a methionine and choline-deficient (MCD) diet (43). Accordingly, Kahraman and colleagues found that liver fibrosis in NASH patients was associated with an increased presence of hepatic NK cells with high NKG2D expression and elevated gene and protein expression of its ligands MICA/B in the liver parenchyma, while in NALFD patients intrahepatic NK cell numbers were reduced (9, 42). The expression of NKG2D in NK cells can be downregulated by factors like TGFβ but activated by cytokines (i.e., IL-2 and IL-15), and ligands including MICA/B in humans and MULT-1, RAE, and HS90 in mice in stressed/damaged lipid-loaded hepatocytes (47, 51, 52) (Figure 1). MICA can be regulated by different immune cells including activated T cells (53) and macrophages in response to TLR activation (54), and its expression was found to be upregulated in NASH but not in NAFLD patients, accordingly to the differential NKG2D expression (42). MICA is a highly polymorphic gene, and a very recent study described the association between different MICA alleles in circulation and pathogenic indicators of NASH vs. NAFLD in the liver. Whether these different alleles could differentially influence NKG2D expression remains undefined.

Overall, even though improved characterization of NK cell markers has revealed distinct cell types, the inability to distinguish between liver-resident and circulating NK cells may explain apparently contradictory results. Indeed, most *in vitro* studies use circulating NK cells from human blood or mouse spleens as few liver-resident NK cells can be purified from a mouse's liver, significantly prohibiting adoptive transfer. The demonstrated differential and tissue-dependent regulation of NK cells highlights the importance of microenvironment in regulation in NK cell function and may explain the contradictory results observed.

## Immunometabolism a Key Determinant in Natural Killer Cells Activity in NASH

In many different pathological conditions activation of immune cells involves metabolic reprogramming toward aerobic glycolysis and a lower reliance on oxidative phosphorylation (OXPHOS) (55). As with other immune cells, metabolic reprogramming of NK cells depends on the type and duration of the stimuli (56), and NK cell-intrinsic metabolic processes influence their performance. While resting NK cells have low basal metabolic rates, i.e., low OXPHOS and glycolysis, they still sustain acute NK cell responses. Under different conditions and in response to particular cytokines, NK cells can either increase reliance on OXPHOS to produce IFNγ and support cytotoxic activity via granzyme production (57, 58), or upregulate expression of nutrient transporters thereby increasing glucose uptake and feeding glycolysis to meet the elevated energy demand (57–60). mTOR plays a key role in this metabolic reprogramming by upregulating glycolysis and overall IFNγ and granzyme B production (57, 59, 60). Cytokines also induce sterol regulatory element-binding protein (SREBP) in NK cells; this is important for controlling glycolysis and OXPHOS via the citrate-malate shuttle (CMS) (not the TCA cycle) independently of its lipid-regulatory function (61). Unlike other lymphocyte subsets, glutaminolysis and the tricarboxylic acid cycle do not sustain OXPHOS in activated NK cells. Glutamine withdrawal, but not the inhibition of glutaminolysis, results in the loss of cMyc protein, reduced cell growth and impaired NK cell responses (62). Activation of NK cells can also be negatively regulated by other factors including transforming growth factor (TGFβ) that suppresses cell metabolism inhibiting glycolysis and OXPHOS (41, 63).

Recent studies have highlighted the role of microenvironment in activation of NK cells and also in how NK cells adapt the expression of nutrient transporters and their metabolism in different tissue compartments. For example, hepatic CD56^bright^ NK cells have lower capacity for glucose uptake but higher for aminoacids as they express low levels of GLUT1 but higher CD98 while CD56^bright^ NK cells in the blood express high levels of GLUT1 and lower levels of CD98 and CD71 (64). Moreover, CD56^dim^ (cytotoxic) are less metabolically active than CD56^bright^ (cytokine IFNγ producing) NK cells (57).

In agreement with the observed influence of microenvironment on NK cells metabolic activity, their metabolic function is also severely disrupted during obesity. Indeed, in obese children with NAFLD, blood NK cells are metabolically stressed, showing increased mTOR, glycolysis and mitochondrial reactive oxygen species (ROS) production that associate with a loss of function (38). In obese adults, NK cells had severely impaired tumor-killing capacity; NK cells took up lipids (via increased CD36) during obesity, leading to profound metabolic alterations (a so-called “metabolic paralysis”) and, ultimately, to a loss of cytotoxic capacity (39). In more detail, lipid-metabolism-related gene expression was altered in lipid-loaded NK cells which showed reduced glycolysis and OXPHOS concomitant with reduced mTOR. These metabolic alterations were confirmed in NK cells from obese patients which was also correlated with impaired proliferation, explaining why fewer NK cells were found in obese patients (9, 36, 38, 39). Lipid-mediated loss of function may lead to tumor development in the context of NASH.

Obesity also promotes the shift of NK cells into an ILC-1 phenotype which is associated with lower cytotoxicity and a reduced capacity to kill tumor cells (41). Cuff and colleagues proposed regulation of mTOR by TGFβ as a mechanism mediating these effects. This could explain discrepancies in the observations of mTOR activation in obese children (with high mTOR) and adults; where low mTOR associates with lower Insulin receptor expression and increased cirrhosis due to lower HSC-killing activity (37).

Overall, both blood and hepatic NK cell activity and metabolism is altered during obesity and insulin resistance, risk factors for NAFLD. Differences in metabolic reprogramming of NK cells associated with NAFLD progression need to be further investigated, as does the therapeutic potential of targeting NK cell immunometabolism for NAFLD treatment.

## The Controversial Role of Natural Killer Cells-Targeting During NASH Progression

Targeting NK cell function in NAFLD has been evaluated using different approaches. For example, mice with impaired cytotoxic capacity (Perforin-KO mice) have reduced liver injury and less fibrosis than controls (35). Absence of IL-15 and IL-15-Receptor alpha in KO mice associated with reduced steatosis that associated with lower inflammation during HFD-induced NAFLD (46). Likewise, the significant protective effects observed in mice lacking TRAIL/TRAIL-Receptor further support the detrimental role of NK cells in NAFLD, via hepatocyte death (65, 66), which also mediated disease progression in genetically induced mouse models of NASH (47, 67) (Figure 1). Finally, mice deficient in NK cells failed to develop fructose-induced NAFLD (68) and depletion of NK cells decreased infiltration of macrophages into the intra-abdominal adipose tissue, reducing inflammation and insulin resistance (69).

Despite these promising indications that targeting NK cells in NAFLD is beneficial, caution is needed. On one hand, several studies have shown that loss of the cytotoxic activity of NK cells during NASH could potentially contribute to the higher susceptibility of obese/NASH patients to liver cancer at later stages of the disease (35, 41). On the other hand, during NASH, NK cells are recruited into the liver via mechanisms including chemokine (C-X-C motif) ligand 10 (CXCL10) (43), where they have an antifibrotic effect (37, 43, 44, 70–74). More specifically, DX5^+^/NKp46^+^ NK cells are activated and contribute to limiting tissue damage and fibrosis during NASH by polarizing macrophages into a proinflammatory M1 phenotype via IFNγ without affecting their cytolytic function (44) (Figure 1). Potent antifibrotic activity of NK cells relies on their ability to kill activated HSC, the main fibrogenic hepatic cell type, via diverse mechanisms including: expression of NKp46 receptor [NCR1 in mice (70)] and its activating ligand (TRAIL) on target cells (74); reduction of Class-I expression; an increased aKIR:iKIR-ratio (72); and relevant metabolic changes including to the PI3K/mTOR pathways (37, 71). Interestingly, the capacity of NK cells to kill HSC seems to be more relevant at early stages of fibrosis while it is impaired at more advanced stages of fibrosis and insulin resistance (37) (Figure 1). Thus, the increase in NK cells frequency, activation and expression of NKG2D and MIC A/B in livers from NASH patients (42) may be effective initially but futile at more advanced stages of disease, overall contributing to the proliferation and activation of HSC. Alternative mechanisms contributing to the deleterious role of NK cells during NASH include: the development of a “myeloid” phenotype of NK cells as these express high levels of CSF1 and CCR2 (40) (Figure 1); and induction of hepatic ER stress via osteopontin, which contributes to insulin resistance in the context of obesity (75). Adipose tissue-resident NK cells also contribute to obesity-induced insulin resistance in adipocytes via IFNγ production leading to macrophage polarization (48, 50, 76).

Overall, the role of NK cells as contributors or modulators of NAFLD progression remains controversial as they have both protective/antifibrotic and deleterious effects during NAFLD.

## Discussion

The presence, regulation and function of NK cells during NAFLD remains controversial. There are several factors that may explain these potentially contradictory results including the only recently improved characterization of NK cell surface markers, and the identification of tissue-specific NK cells. Indeed, better characterization of NK cells using distinct intra- and extra-cellular markers and metabolic indicators, has revealed previously unknown distinct characteristics of peripheral, liver, and adipose-tissue NK cells; all are relevant to progression of NAFLD to NASH and liver fibrosis (34, 35, 38, 39).

Disease stage also greatly influences activation of NK cells, which negatively correlates with fibrosis level in the liver. Thus, apparent contradictory outcomes may reflect the differential function of NK cells at different stages of disease; active and beneficial at early stages of fibrosis but becoming detrimental upon losing anti-tumor capacity thereby contributing to tumor progression at later stages (37) (Figure 1).

To date, there have been attempts to generate NK cell-targeted therapeutic approaches to treat NAFLD/NASH focused on their inhibition/depletion. Studies utilizing genetic targeting of total NK cells [IL6Ra and Cfs1 (40), E4bp4 (48), and NCR1 (50)] have shown promising reduction of obesity, liver steatosis, and improved insulin signaling in mice fed with HFD (50, 69). Also, antibody-mediated depletion of NK cells (NK1.1 and GM1) in HFD-fed mice correlated with lower infiltration of macrophages in adipose tissue and improved systemic insulin signaling but had no effects on reducing weight gain (48, 50). Nonetheless, the heterogeneity of NK cells, as well as the great influence of the microenvironment on controlling their functions, underlines the difficulty of developing a unique global strategy of NK cell depletion to successfully treat NAFLD/NASH.

Increasing evidence support the influence of metabolism in controlling NK cells and therefore the therapeutic potential of targeting metabolic regulators during NAFLD/NASH. These may include the use of mTOR inhibitors that have shown to efficiently reduce the cytotoxicity of NK cells (59, 60). However, this may have a detrimental impact at later stages of NASH progression, contributing to tumor development. In addition, the complex regulation of mTOR expression during NAFLD/NASH, changing at different stages of the disease; increased in children (38) but reduced in adults (39) difficult the targeting of this pathway as an effective approach to treat disease progression.

Targeting of other factors that regulate NK cell activation by influencing metabolism have also been proposed. i.e., TGFβ represses the expression of nutrient receptors, glycolytic capacity and OXPHOS overall reducing NK cell cytotoxicity, supporting that inhibiting TGFβ could restore NK cell function during NAFLD/NASH (63, 77).

Still, we lack in depth understanding of the role of metabolism in regulating liver tissue-resident NK cells that may be particularly susceptible to the extreme changes in metabolic conditions typical in the liver. This notion is supported by recent studies demonstrating the role of microenvironment in determining NK cell activity, particularly metabolism (57, 64), which may differentially influence the activation of NK cells in different organs. Future research in this direction will improve our knowledge on the tissue-specific metabolic pathways regulating NK cell activity, paving the way for the development of therapeutic strategies targeting NK cell metabolism to treat NAFLD/NASH.

In summary, the potential of therapeutic approaches targeting NK cell metabolism in NAFLD will greatly benefit from better characterization of NK cells; resident vs. recirculating vs. blood. This would enable improved targeting strategies that account for the distinct tissue microenvironment and unique metabolic characteristics.

## Author Contributions

All authors listed have made a substantial, direct and intellectual contribution to the work, and approved it for publication.

## Conflict of Interest

The authors declare that the research was conducted in the absence of any commercial or financial relationships that could be construed as a potential conflict of interest.
